# Acceptability, Usability, and Clinical Integration of a Clinic-Based Digital Game for HPV Education: Qualitative Perspectives from Adolescents, Parents, and Healthcare Providers

**DOI:** 10.3390/vaccines14020116

**Published:** 2026-01-26

**Authors:** Elizabeth Reifsnider, Satya Subedi, Nouran Ghonaim, Megan Whaley, Angela Chia-Chen Chen

**Affiliations:** 1 Ellmer College of Nursing, Old Dominion University, Virginia Beach, VA 23453, USA; ereifsni@odu.edu; 2 College of Nursing, Michigan State University, East Lansing, MI 48824, USA; subedisa@msu.edu (S.S.); ghonaimn@msu.edu (N.G.); whaleym1@msu.edu (M.W.)

**Keywords:** HPV vaccination, human papillomavirus, serious games, digital health intervention, adolescent health, parent engagement, vaccine hesitancy, healthcare providers, clinic-based intervention, qualitative research

## Abstract

**Background/Objectives:** HPV vaccination is safe, effective, and recommended at ages 11–12, yet uptake remains suboptimal. Serious video games may offer an innovative strategy to deliver brief, engaging education during clinic visits. This qualitative paper, embedded within a mixed-methods study, examined adolescents’, parents’, and healthcare providers’ (HCPs’) perceptions of the acceptability, usability, and perceived clinical applicability of HPV Detective, a tablet-based digital game designed to provide HPV-related education to parent–child dyads during pediatric clinic wait times. **Methods:** Eight adolescent–parent dyads (N = 16) and three HCPs from university-affiliated pediatric clinics participated in 30–60-min semi-structured Zoom interviews. Interviews were audio-recorded, transcribed, and thematically analyzed by two coders, with discrepancies resolved by consensus and reviewed by a third researcher. **Results:** Participants identified five key dyadic themes and four HCP themes. Adolescents described the gameplay as intuitive and enjoyable, highlighting interactive challenges and realistic avatars. Parents valued the clarity of HPV information and noted that the game helped initiate health-related conversations. Both adolescents and parents suggested enhancements including voice narration and greater customization and agreed that the game was well suited for 10–15-min clinic wait times, with text messaging preferred for follow-up. HCPs emphasized challenges such as parental hesitancy and competing clinical demands and viewed the game as a feasible adjunct to support vaccine-related discussions. **Conclusions:** Findings suggest the acceptability, usability, and perceived clinical applicability of a brief, clinic-based digital game for HPV-related education and engagement among adolescents and their parents.

## 1. Introduction

Human papillomavirus (HPV) is the most common sexually transmitted infection in the United States and a leading cause of cervical, oropharyngeal, and other anogenital cancers [1]. The HPV vaccine provides safe, effective, and long-lasting protection against cancers caused by HPV infections [2]. However, national vaccination coverage remains below target levels—only 62.9% of U.S. adolescents aged 13–17 years were up to date with the HPV vaccine series in 2024 [3], falling short of the Healthy People 2030 objective to *increase the proportion of adolescents who get recommended doses of the HPV vaccine by age 13–15 years to 80%* [4]. Recent evidence synthesis by Ou et al. [5] has highlighted multiple, persistent barriers that continue to impede HPV vaccination among youth. In a national survey conducted in the United States, Szilagyi et al. [6] found that nearly one quarter of parents of adolescents questioned the safety, effectiveness, or benefits of the HPV vaccine; adolescents of vaccine-hesitant parents were about one-third less likely to be vaccinated, and approximately 20% of parents did not agree that the vaccine was beneficial for their child or that they should follow their healthcare provider’s vaccination recommendations. Similar patterns have been observed internationally; in Shanghai, China, HPV-specific vaccine hesitancy was reported by approximately 26–28% of parents of adolescents, compared with about 9–11% reporting general vaccine hesitancy [7]. - In a U.S.-based trend analysis, Adjei et al. [8] further identified common parental reasons for HPV vaccine hesitancy, including perceptions that vaccination is unnecessary due to lack of sexual activity, absence of healthcare provider recommendation, limited knowledge about HPV, and concerns about vaccine safety. Together, these findings underscore the need for developmentally appropriate, context-sensitive interventions that support parent–adolescent decision-making and can be feasibly integrated into routine clinical care.

At the individual and family level, low knowledge about HPV, concerns about vaccine safety, and limited parent–child communication reduce motivation and informed decision-making [5,6,7,8,9]. At the provider and system level, inconsistent provider recommendations, missed opportunities during clinic visits, and logistical challenges such as scheduling difficulties hinder completion of the vaccine series. Societal barriers, including stigma surrounding sexual health, cultural discomfort, and misinformation further reinforce hesitancy and mistrust. These multilevel barriers underscore the need for innovative approaches that actively engage adolescents and parents in real-world healthcare encounters to promote vaccine confidence and uptake.

Digital serious video games (SVGs) offer a promising and scalable strategy for health promotion. SVGs combine entertainment with educational and behavior-change elements grounded in Social Cognitive Theory [10,11,12] and persuasive technology [13]. Through interactive and experiential learning, SVGs can enhance attention, motivation, and knowledge retention [14,15,16,17]. A recent systematic review by Ou et al. [5] suggested that game-based interventions can improve vaccine-related knowledge, attitudes, and behavioral intentions among youth, particularly when integrated within existing healthcare delivery systems. Yet few studies have examined these interventions within actual pediatric clinic workflows, and even fewer have incorporated both parent and adolescent perspectives. To address gaps in HPV vaccine uptake among adolescents, we conducted a mixed-methods randomized controlled trial (RCT) evaluating HPV Detective, a tablet-based educational game designed to promote HPV vaccination among parent–adolescent dyads in pediatric clinics.

This paper presents qualitative findings from interviews with parent–child dyads and healthcare providers (HCPs) conducted as part of a larger mixed-methods project evaluating a digital HPV educational game. This qualitative component examined adolescents’, parents’, and HCPs’ perceptions of acceptability, usability, and perceived clinical applicability and did not assess HPV vaccination effectiveness or uptake, which are evaluated in the parent randomized controlled trial. Dyadic interviews enabled simultaneous exploration of both adolescent and parent experiences, providing insights into how the intervention facilitated communication, shaped perceptions, and influenced vaccination decision-making within families. Including HCPs offered complementary perspectives on the feasibility of integrating the game into clinical workflows and identified facilitators and barriers to adoption in real-world practice. Together, these perspectives offer a comprehensive understanding of end-user engagement and implementation considerations, informing refinement of the intervention and guiding strategies for broader dissemination.

## 2. Materials and Methods

### 2.1. Design and Setting

The qualitative interviews were embedded within a mixed-methods randomized controlled trial (RCT) evaluating HPV Detective, a brief digital educational game designed to support HPV vaccine–related education during pediatric clinic visits. The main trial used a parallel-group design in which eligible parent–child dyads were randomized, after completion of baseline surveys, to either the HPV Detective game intervention or a usual care comparison condition. The main trial was designed to evaluate feasibility and acceptability of the intervention, as well as preliminary effectiveness related to HPV vaccine uptake among adolescents, alongside theoretically informed mediators (e.g., knowledge, attitudes, parent–child communication, and decision-making processes) and potential moderators (e.g., child sex and age). In contrast, the qualitative component reported here aimed to provide in-depth insight into the intervention’s acceptability, usability, and perceived clinical applicability.

We collaborated with four university-affiliated pediatric clinics that serve families facing barriers to care, including limited health education and insurance coverage, poverty, language barriers, and legal challenges. In this study, “vulnerable children” refers to pre-adolescents and early adolescents experiencing barriers that limit timely, culturally and developmentally appropriate vaccine education. The study protocol was approved by the Michigan State University Institutional Review Board, and written informed consent and assent were obtained from all participants prior to data collection.

### 2.2. Participants and Eligibility

#### 2.2.1. Adolescent–Parent Dyads

Adolescents were eligible if they (1) were 11–14 years old; (2) had not received the first HPV vaccine dose; (3) at approach, reported that they and their parent/legal guardian either did not plan to vaccinate or were undecided; and (4) spoke and read English. The target age range aligns with national recommendations to initiate HPV vaccination at ages 11–12, when cancer prevention is maximized, and a 2-dose schedule applies if the first dose is received before age 15 [1,2]. Parents/legal guardians were eligible if they: (1) were the parent or legal guardian of an enrolled child; (2) owned a smartphone; (3) agreed to participate in research activities at clinics; and (4) consented to receive study-related text messages. In families with multiple eligible children, the oldest child who had not initiated HPV vaccination was recruited.

#### 2.2.2. Healthcare Providers (HCPs)

Licensed HCPs were eligible if they (1) were ≥20 years old and provided care to children at participating clinics; and (2) spoke and comprehended English.

### 2.3. Recruitment and Procedures

Recruitment flyers were distributed through partnering clinical sites, university listservs and newsletters, local community organizations, libraries, social media platforms (e.g., Meta and WhatsApp), and word of mouth. Community-based recruitment also occurred through attendance at local community events (e.g., health fairs and family-focused outreach events), where study team members shared information about the project and distributed recruitment flyers. The research team also conducted in-person recruitment at partnering clinics. Clinic staff referred potentially eligible individuals during their appointments to the research team, who then provided a flyer introducing the project. Interested individuals completed an eligibility survey by scanning a QR code or entering the URL provided on the flyer.

Families who met the eligibility criteria were contacted to schedule a virtual meeting with the study team to confirm eligibility, review study procedures, assess interest, and address any questions. Following this meeting, participants received a secure link to complete electronic consent/assent and baseline surveys (T0) for both the parent and child. After completing the baseline surveys, families were randomized to either the intervention or comparison group. For families who declined participation, de-identified reasons for nonparticipation were documented.

### 2.4. Intervention

The HPV Detective game intervention was developed using stakeholder input from clinicians, parents, and youth [18] and integrates principles of Social Cognitive Theory [10,12] and persuasive technology [13] to build self-efficacy, enhance motivation, and support parent-youth communication and family-provider engagement around HPV vaccination. The game includes an introductory tutorial, point-collection challenges, and interactive knowledge scenarios designed for both boys and girls. The educational content emphasizes HPV-related cancers and prevention benefits for both sexes, while addressing common misconceptions about HPV risk, transmission, and sexual behavior [18].

The game was delivered on a tablet and designed to be completed within approximately 15 min to align with typical clinic wait times. Children were the primary players, and parents were instructed to sit alongside them during gameplay to facilitate parent–child communication. Parents were thus able to view and engage with the educational content delivered through the game interface in real time. Tablets, rather than smartphones, were used to ensure a screen size adequate for shared viewing by both participants, consistent with recommendations from the family advisory board in the pilot research [18].

**Figure 1:**
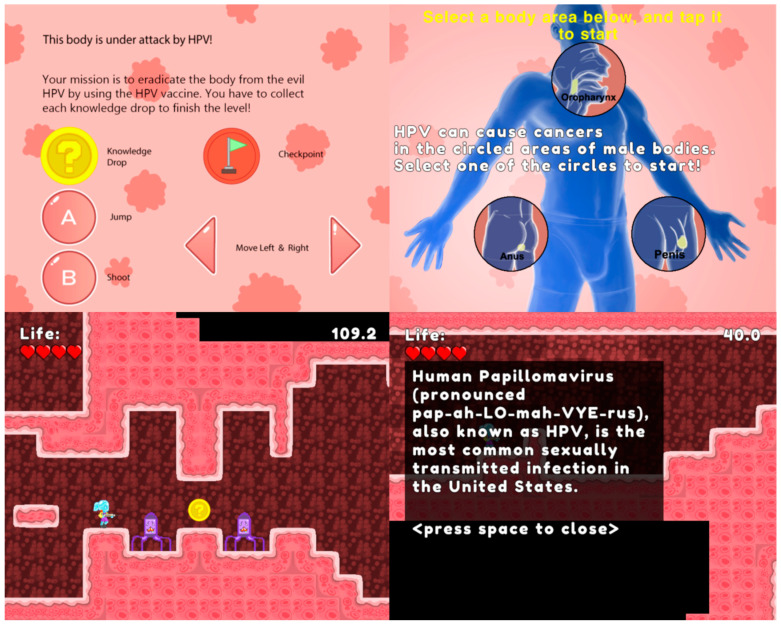
HPV Detective Game Avatar, Graphics, and Gameplay Elements.

Representative screenshots illustrating key game features (e.g., avatars, graphics, and gameplay elements) are provided in Figure 1. Because the HPV Detective game is still under active testing, public access to the full intervention is restricted; however, a tutorial video demonstrating core game features and content, which was used with parent–child dyads during the study, is available at https://mediaspace.msu.edu/media/HPV+Detective+Game+Tutorial/1_5wl5imnu (accessed on 13 January 2026).

### 2.5. Qualitative Interviews

Semi-structured interviews were conducted with a purposive subset of participants from the intervention arm of the main randomized controlled trial following completion of the intervention. Of the 31 parent–child dyads randomized to the intervention condition, eight dyads were identified and invited to participate in qualitative interviews after completion of the intervention. Dyads were purposively selected to ensure variation by youth sex (boy/girl) and age (11–12 vs. 13–14), consistent with the exploratory aims of the qualitative analysis.

Interviews elicited adolescent and parent perspectives on engagement with the intervention during the clinic visit, perceived usability and acceptability of the game, and its influence on parent–child communication and decision-making. We also interviewed three HCPs from participating study sites (two pediatricians and one nurse manager) to explore feasibility, workflow fit, and perceived facilitators and barriers to integrating this game into routine care.

Interviews were conducted via Zoom using semi-structured interview guides tailored for adolescents, parents, and HCPs and lasted 30–60 min. Interviews were conducted by three trained members of the research team, including the study’s principal investigator and two research assistants. Interviewers received study-specific training led by the principal investigator, which included review of the interview guides, mock interviews, and discussion of strategies for engaging adolescents, parents, and HCPs in qualitative interviews. Interviewers were not involved in participants’ clinical care and had no prior relationships with interviewees. Interview roles were defined in advance, with one team member leading the interview and another available to take observational notes and provide technical support as needed. The principal investigator reviewed early interviews to ensure consistency and adherence to the interview guides. With written permission, all interviews were audio-recorded and professionally transcribed. Each dyad and HCP received a $30 incentive for participation.

Sample size was guided by principles of thematic saturation. Given the focused aims of the qualitative inquiry, the shared intervention experience among participants, and the depth of the semi-structured interviews, the sample of eight parent–child dyads was sufficient to identify recurring themes related to acceptability, usability, and perceived clinical applicability. Across interviews, no substantially new themes emerged in later dyad interviews. Similarly, interviews with three HCPs were sufficient to capture key perspectives related to feasibility and workflow integration across participating clinics.

### 2.6. Data Analysis

Two researchers conducted inductive thematic analysis following standard steps [19]: familiarization, initial coding, theme development, theme review/refinement, theme definition/labeling, and synthesis of findings. A third researcher reviewed the codebook, coded excerpts, and thematic outputs; discrepancies were discussed and resolved by consensus. COREQ guidelines [20] were followed to enhance transparency and reporting quality.

### 2.7. Rigor and Trustworthiness

Rigor was guided by established qualitative standards [21,22]. We emphasized openness (approaching data without preconceptions), questioning pre-understandings, and maintaining a reflective stance (reflexivity). Credibility was supported through triangulation of perspectives (adolescents, parents, HCPs), shared codebook development with clear definitions and structure, iterative team debriefs, and transparent documentation. Transferability was enhanced by providing rich descriptions of the participants, their environments, their personal characteristics, and in some cases, their views on vaccination and vaccine hesitancy.

## 3. Results

The findings presented below reflect participants’ perceptions of the game’s acceptability, usability, and perceived clinical applicability, rather than objective measures of vaccination behavior or effectiveness. Table 1 summarizes the qualitative coding framework, mapping representative codes to subthemes and overarching themes across participant groups.

### 3.1. Participant Characteristics

Eight parent–adolescent dyads and three HCPs participated in the qualitative interviews. Adolescents were 11–14 years old (mean = 11.9); four identified as male and four as female. Parents, most of whom were female, were 38–48 years old (mean = 43.3) and identified as White non-Hispanic (n = 5), Asian American (n = 2), or Hispanic/Latino (n = 1). The HCPs included two male pediatricians with 11–20 and >21 years of experience and one female nurse manager with >21 years of experience overseeing three pediatric clinics. The participating clinics primarily served underinsured and multilingual populations, with English and Spanish being the most commonly spoken languages.

### 3.2. Parent–Child Dyads Perspectives

Five key themes emerged from the dyadic interviews. Participant quotes are labeled by role (C = child, P = parent, HCP = healthcare provider) and original study identification number assigned at enrollment in the parent trial (N = 65 dyads). The qualitative sample consisted of eight dyads recruited from the intervention group (n = 31), and original IDs were retained to preserve linkage with the parent dataset. Common themes across dyadic interviews included the following.

### 3.3. Avatar Realism and Representation

The players stated that avatars enhanced their playing of the HPV game. The adolescents identified avatars as realistic as far as the sex of the player and wanted them to have more personality and have speaking ability. Some players stated that there was too much pixelation of the avatars in the game. There were differing opinions on the speed of advancement in the game; some adolescent players believed that the game advanced too slowly and some wanted more time to read the statements in balloon form from the avatars. They also wanted more colors in the avatars. Participants consistently described how the avatars shaped their engagement with the game, and replied positively when asked about the use of the avatars in the games when questioned about the appropriateness of avatars and their contribution to the game: reflected in comments such as:

“It looks real. It was a good thing.” (C043)

“There should be more colors” (C045)

“I don’t remember the colors. It was fine. I think about the colors.” (C046)

“Avatar was pretty nice. I liked the avatar.” (C051)

### 3.4. Game Recommendation to Others

The players wanted their family and friends to participate in the game and asked if they could share the link to the game with family and friends. They also validated the quality of the game as something other adolescents would enjoy. Participants reported that video games were an effective and engaging way to learn about HPV. Although some had previously learned about HPV from different sources, the majority indicated that the information they received was limited or incomplete:

“Like, in health class, we have health class. They teach us a little bit about it in health class. Not that much.” (C046)

During gameplay, participants identified and clarified misunderstandings stemming from prior information from various sources and expressed appreciation for learning accurate information through the HPV game. Parents expanded on this theme by expressing positive perceptions of the game and their willingness to share the game with others:

“We would recommend [it]…it’s good place to start if you’re in question or you’re on the fence outside of just them saying you should do this or do you want to do this, like it gives you a little bit of information and then it allows you then to do more investigation to then be able to ask more questions” (P032)

“It’s a good game that he enjoyed it, so I think it’s a nice game” (P045)

“[Will recommend to others] Very likely because they don’t know a lot about that.” (P046)

Adolescents reported that they were willing to share the game with friends if asked but were reluctant to initiate conversations about HPV or actively promote the game to peers. As one participant explained:

“When you ‘re just talking to your friend, it’s kind of random to be like you should go play this, you should go play this game about HPV, so that’s something I would definitely tell them if they asked, but otherwise it’s kind of, yeah, random” (C004).

### 3.5. Enjoyment and Engagement in Gameplay

Participants described the game as enjoyable and attention-holding, highlighting its entertainment value and engaging features. Adolescents noted that the game format made learning about HPV more appealing and increased their willingness to engage with the content. Several participants suggested potential enhancements to sustain engagement, including adding more levels, increasing difficulty, and incorporating periodic quizzes. While the game was generally viewed as age-appropriate for children and families, some older adolescents indicated a preference for a faster-paced and more challenging experience. Participants also expressed mixed views about content scope, with some noting overlap with prior school-based instruction and others describing prior misunderstandings about HPV. A small number of parents raised concerns about specific game elements, such as scenes perceived as intense when characters fell into a pit following incorrect choices. Overall, participants emphasized the game’s entertainment value and interactive mechanics, as illustrated by the following quotes:

“[Best way to counter vaccine lack of knowledge] I don’t know. I feel like making it a game was good because then I kinda like it makes people more inclined to, like actually do it because it’s a fun game.” (P051)

“[What they liked the most] Like I like all of it.” (C043)

“Like the, when you like, you have the thingy that shoots the other guy.” (C046)

“I like the enemies and I like blasting them.” (C051)

### 3.6. Visual Appeal and Color Contrast

Participants generally viewed the game’s visual design positively, noting that the fonts and graphics were acceptable and that the use of color was appealing. However, some participants observed inconsistencies in visual presentation, describing areas that appeared as black or empty spaces or seemed visually out of place compared with the rest of the game. Several players—particularly older adolescents—also commented on the pacing of gameplay, noting that movement and progression felt slow and that on-screen text advanced gradually. While brightness and contrast were not widely perceived as problematic, a small number of participants suggested that greater contrast could enhance visibility. Some participants also expressed a preference for increased use of voice narration and reduced reliance on text. These perspectives were reflected in the following comments from parents and adolescents:

“Yeah, I liked the colors I noticed, like their ground was like a color. But then there were some parts where there was just black void.” (P051)

“So like one or two spots seemed a little out of place because they didn’t match the rest.” (P051)

“Yeah, but it was a little slow. Like the movement was a little slow and the gravity was also a little slow… Things didn’t happen fast.” (C051)

“I don’t think it was too bright or too dark.” (C043)

### 3.7. Tutorial Usability and Suggested Improvements

Adolescents described the tutorial as generally intuitive and sufficient for understanding how to begin playing the game, indicating that it supported initial onboarding and navigation. While most participants found the tutorial easy to understand, some characterized it as only moderately helpful. Feedback focused primarily on the tutorial’s audio narration; several participants described the voice as robotic or unnatural and expressed a preference for a more human-sounding narrator. These perspectives were reflected in the following comments:

“[The tutorial was] sort of [easy to understand]” (P045)

“Easy [to understand]” (P046)

“OK, yes, it was easy to understand” (P051)

“I didn’t like the voice.” (C043)

“It’s kind of weird…like a robot” and “Yeah [would prefer human voice]” (C046)

In summary, participants viewed the game as a useful and acceptable tool for health education, noting its potential to clarify misunderstandings about HPV that arose from prior information obtained from different sources and to support discussion and further information-seeking. Both parents and adolescents indicated that they would recommend the game to others and agreed that it was age-appropriate and well suited for brief use during clinic wait times prior to seeing a healthcare provider. Participants also expressed a preference for text messaging as a follow-up modality.

Adolescents appreciated the intuitive gameplay and interactive features such as the “blaster,” although they were more likely to share the game if asked than to initiate conversations about HPV. Participants described avatars as generally realistic and contributing to engagement, and suggested several refinements to further enhance the design, including richer color use, clearer and more consistent visuals, faster pacing, additional levels or challenges for older adolescents, incorporation of quizzes to reinforce learning, and improved voice narration.

### 3.8. Healthcare Providers (HCPs) Perspectives

The HCPs, although fewer in number, offered rich insights into the HPV Detective game, the characteristics of their patient populations, challenges of pediatric practice in the current political and healthcare environment, and recommendations to improve HPV vaccination rates among adolescents. Direct quotes were labeled using participant type and study ID (e.g., HCP1 = healthcare provider #1). Four key themes emerged from HCP interviews.

### 3.9. Diverse and Vulnerable Patient Population

HCPs described caring for highly diverse patient populations experiencing structural vulnerability, characterized by high rates of underinsurance, reliance on Medicaid, and intersecting social determinants of health that complicate access to preventive services such as HPV vaccination. Many families faced additional challenges, including mental health concerns. English was the most commonly spoken language, followed by Spanish; however, providers also reported working with families who spoke more than five additional languages. Cultural backgrounds varied widely, with some groups demonstrating greater receptivity to vaccination. Providers noted that cultural concordance between HCPs and families sometimes facilitated trust and eased vaccine-related communication and decision-making. These perspectives were reflected in comments such as:

“We probably have more than 50% of population on Medicaid…after health department we are probably a second Practice with the most Medicaid.” (HCP 1)

“…Mostly English…[families] from Southeast Asia and China. Families speak Arabic and Spanish. Indian languages, [Chinese] Mandarin and Arabic, those are three top ones I would say.” (HCP 3)

“…if the physician is from the same culture or the background, I think it carries a little bit more weight probably. I think they tend to have a trust…it helps in that way because even for flu vaccines, a lot of Indian population don’t get flu vaccines…we talk about the reasoning behind the flu vaccine and then they then they tend to get it soon.” (HCP 3)

### 3.10. Range of Pediatric Healthcare Needs

The reasons for visits included routine checkups based on age, as well as ongoing chronic diseases such as diabetes and asthma and acute illnesses such as ear infections and strep throat. Children with medical complexities, such as those with genetic or congenital conditions or preterm birth, are also commonly seen. Their comments illustrated the clinical complexity of the populations they serve:

“There’s a lot of patients with medical complexity…children who are born prematurely…kids with genetic conditions.” (HCP1)

“So, a lot of our business is well-child care…a big population is going to be mental health concerns…ear infections, strep throat, rashes, common colds, minor injuries, asthma. We manage like basic very straightforward cases, but if it’s anything that’s more complex [we refer].” (HCP 2)

### 3.11. Pro-Vaccine Practice and Parental Hesitancy

The three branches of the practice all endorsed a pro-vaccine practice. The practice would accept patients who were not vaccinated but parents were counseled at every visit on the importance of vaccination and were asked to sign vaccination refusal paperwork. The HCPs had received complaints from parents about their policy of pro-vaccination. Parents who were from developing countries tended to be more receptive to vaccinating their children. HPV vaccination was routinely offered beginning at age 11, though some parents hesitated due to discomfort discussing sexual activity or uncertainty about HPV risk. HCPs noted that vaccination rates declined during the COVID-19 pandemic and had not returned to pre-pandemic levels. They also observed that parents were often more receptive when HPV vaccination was framed as cancer prevention for their children and future partners. Providers described these varied sources of hesitation and their strategies to address them, reflected in comments such as:

“…HPV rate went down during pandemic…people [adolescents] didn’t feel comfortable at that time until 2022 and beginning of 2023 to come back… Some people don’t, you know, don’t believe in vaccines…I think it’s even more important for us on ground to continue explaining and emphasizing the importance of vaccines and prevention” (HCP 1).

“I have a few families who still have doubts about HPV vaccination for certain reasons. Because in many cultures you know, getting sexually active is not something that they talk about, and I think the one of the reasons for HPV vaccination is you give the vaccination before you get sexually active…that concept is still pretty new to them.” (HCP 3)

### 3.12. Digital Tools and Workflow Integration

HCPs reported recommending reputable online resources, such as American Academy of Pediatrics materials, to parents and expressed openness to using digital tools, including online and computer games for health education. They were cautious about integrating the HPV game into routine clinical practice due to concerns about potentially disrupting clinic flow, particularly when families arrived late for appointments. Despite these reservations, providers acknowledged that with appropriate adjustments, the game could serve as a useful educational tool to support vaccine discussions. One HCP compared the process to the initial implementation of developmental screening tools, which had temporarily disrupted workflow but eventually became well integrated into clinic procedures. HCPs explained both the opportunities and workflow considerations surrounding digital tools, noting:

“When we started [screening tools such as PHQ2], it was much more hesitancy on kind of why we’re doing this. It’s still some hesitancy, but much less…when it gets kind of implemented and we see results that we can do about this…we can address this early and be preventing real problems.” (HCP 1).

“They [kids] like the little games or whatever on the little tablet that they can play while they’re waiting. That would be a good idea…I can see most of the kids coming in and like just gravitating towards like a tablet with a game on it.” (HCP 2)

“It would be something like that you could send to the parents’ phone and they could do it with their child? Like even after they leave or before they come in or while they’re waiting for the doctor. It could be if it was easy like an app or something where they’d be willing to download it. Then I think that would be a lot more feasible.” (HCP 2)

“It just depends on the timeline because many families also come late to the appointment. They are already late and on top of that, if they are playing that eight to 10 min, it adds up.” (HCP 3).

## 4. Discussion

The qualitative interviews, conducted as part of a parent RCT, explored adolescents’, parents’, and HCPs’ perceptions of acceptability, usability, and perceived clinical applicability of a brief clinic-based digital game. The findings provide important insights into how these key stakeholders perceived the HPV Detective game and its potential role in HPV-related education and engagement during clinical encounters. The parents and adolescent participants described the game as informative, engaging, easy to understand, and well suited for brief waiting periods prior to seeing their healthcare providers. HCPs similarly perceived the game as a potentially useful adjunct for addressing commonly reported challenges in HPV vaccine discussions, such as parental hesitancy, limited visit time, and cultural or linguistic barriers documented in prior research [23,24]. HCPs emphasized that a short, engaging digital tool could help structure or initiate vaccine-related conversations during the clinical visit. Our findings complement prior literature suggesting that serious video games can support vaccine-related knowledge and attitudinal processes [5,25], while extending this work by highlighting how adolescents, parents, and healthcare providers perceive the acceptability and usability of such tools within real-world clinical contexts.

Adolescents in our study described the gameplay as enjoyable, intuitive, and visually appealing, echoing prior work showing that well-designed game mechanics support immersion and learning [26]. Parents similarly appreciated the clarity and simplicity of the educational content, consistent with research indicating that parents prefer concise HPV information that reduces confusion and supports comprehension [27]. Adolescents’ willingness to recommend the game to peers suggests receptivity to interactive, technology-based health tools, consistent with prior research demonstrating strong youth engagement with game-based vaccine and health interventions [5,25,28].

Findings from this qualitative study align with prior research suggesting that interactive, game-based approaches are acceptable and engaging tools for vaccine education among youth and families. Previous studies indicate that serious games can support vaccine-related learning, engagement, and discussion by addressing knowledge gaps and misinformation, without necessarily demonstrating behavioral effects. For example, Cates et al. [25] reported that a serious video game designed for preteens was feasible to implement, well accepted by both children and parents, and perceived as a helpful tool for facilitating conversations about HPV vaccination. Similarly, Hopkins et al. [28] described the co-design of a mobile-based vaccine game that was engaging and culturally acceptable and improved vaccine knowledge and resistance to misinformation among youth and community members. Extending this work to other vaccine contexts, Ou et al. [5] demonstrated that a game-based intervention targeting COVID-19 vaccination improved vaccine knowledge and addressed misinformation, highlighting the potential of interactive games to target key drivers of vaccine hesitancy. Together, these studies provide context for interpreting the acceptability, perceived utility, and implementation considerations observed in the present qualitative analysis. Additionally, healthcare providers’ emphasis on persistent organizational barriers such as competing clinical priorities and missed opportunities during clinic visits supports the need for innovative approaches like digital games to supplement vaccine counseling in busy clinical environments.

At the same time, several findings extend beyond or differ from what has been reported in prior literature. Earlier studies of digital and game-based health interventions frequently highlight usability challenges, including confusing navigation, unclear instructions, and excessive cognitive load that can overwhelm young users and reduce engagement [17,29,30,31]. In contrast, both adolescents and parents in our study consistently described the HPV Detective game as easy to understand, straightforward to navigate, and appropriately paced. This suggests that the game achieved a level of intuitive design that is not always observed in similar health-focused digital tools. Furthermore, whereas most HPV-related game research has concentrated on knowledge improvement, our participants devoted notable attention to aesthetic and experiential elements such as avatar realism, graphical clarity, voice narration, and color contrast, which have received limited discussion in prior evaluations of HPV educational games. These insights reflect contemporary user expectations shaped by mainstream gaming environments and underscore the importance of production quality for maintaining credibility and engagement, especially among adolescents. Finally, healthcare providers in our study offered detailed perspectives on workflow integration, highlighting practical concerns such as appointment timing, patient lateness, and competing clinical demands. Such operational realities are rarely addressed in prior HPV gaming studies. These novel insights help clarify how digital tools can be realistically incorporated into pediatric clinical settings and identify the workflow adaptations necessary for successful adoption.

This study has several strengths. By incorporating perspectives from adolescents, parents, and healthcare providers, it offers a comprehensive understanding of the HPV Detective game’s usability and potential for clinical integration. The qualitative design allowed for in-depth exploration of user experiences, and the focus on real-world pediatric clinic workflows addresses an implementation gap rarely examined in prior HPV gaming research. By engaging parents alongside adolescents in gameplay and HPV-related learning, the intervention targets key drivers of parental vaccine hesitancy—such as limited knowledge, safety concerns, and lack of confidence—that prior studies have shown to impede HPV vaccination uptake among children [8]. Game-based approaches have been shown to support engagement, learning, and vaccine-related discussion, offering a promising strategy for addressing these barriers [5,25,28].

However, the small, purposive sample drawn from university-affiliated clinics may limit the transferability of findings to broader youth and parent populations or to healthcare systems in other countries with different clinical workflows, cultural contexts, and vaccination policies. Participants may also have been more open to health interventions or digital tools than the general population, which could further limit generalizability. Finally, findings reflect participants’ self-reported perceptions and experiences rather than objective vaccination behaviors or outcomes; therefore, conclusions regarding the game’s impact on HPV vaccine uptake cannot be drawn from this qualitative study and require confirmation through quantitative analyses from the main trial.

Although this qualitative study focused on delivery within pediatric clinic settings, participants’ emphasis on brief, engaging, and developmentally appropriate content suggests that HPV Detective may also be adaptable to other settings where adolescents routinely receive health education, such as schools. School-based delivery could offer a complementary approach for reaching adolescents who experience missed preventive care visits or face access barriers in clinical settings. Importantly, participants’ perspectives on the acceptability, usability, and perceived clinical applicability of the game underscore the relevance of theoretically informed mediators such as knowledge, attitudes, parent–child communication, and shared decision-making, which can inform the selection and interpretation of objective outcome measures in the main randomized controlled trial and future studies.

From a public health perspective, these findings contribute to understanding how digital game–based interventions may complement existing HPV vaccination strategies by supporting early, developmentally appropriate education and facilitating communication among adolescents, families, and HCPs. Rather than functioning as stand-alone interventions, interventions such as HPV Detective game may serve as supportive components within broader, multilevel public health approaches aimed at reducing missed educational opportunities and promoting equitable access to vaccine information. Qualitative insights into acceptability, usability, and clinical workflow fit are particularly relevant for informing how digital tools might be integrated into coordinated prevention strategies across clinical, school, and community settings.

## 5. Conclusions

This qualitative component indicates that a brief, clinic-based digital game intervention is a feasible and acceptable approach for delivering HPV-related education to adolescents and their parents within pediatric care settings. Engaging adolescents and parents before the clinical encounter—whether in waiting rooms or exam rooms—provides a practical opportunity to deliver concise HPV education without substantially disrupting clinic flow. HCPs viewed the HPV Detective game as a useful educational tool for initiating conversations about HPV-related information, including cancer prevention, during clinical encounters.

The findings highlight the potential of interactive digital tools to enhance HPV-related education and engagement among adolescents and families in pediatric settings. Future iterations of the game should incorporate improved audio quality, expanded personalization features, greater avatar diversity, and multilingual options to better serve diverse patient populations. Further research with larger and more diverse samples is needed to assess generalizability, and quantitative longitudinal studies are warranted to examine vaccination-related outcomes.

Game-based interventions offer a scalable and innovative strategy to support HPV vaccine education, and these findings can inform implementation efforts across varied healthcare environments. Overall, these qualitative findings contribute implementation-relevant insight into how serious games may be incorporated into pediatric clinical settings to address educational gaps and support communication about HPV vaccination. As an interactive and engaging modality, serious games can convey complex health information in an accessible and unobtrusive manner and may be adapted across a wide range of health promotion, health protection, and disease prevention contexts.

## Figures and Tables

**Table 1:** Codes, Subthemes and Themes.

**Children and Parents**			
	**Theme**	**Subtheme**	**Representative Codes**
	Tutorial Usability	Tutorial clarity and onboarding	Tutorial; easy to understand; confusing; suggestions for improvement
		Follow-up preferences	Text messaging; email; phone calls; reminders from healthcare office
		Incentives	Digital gift cards; physical gift cards; cash
		Post-game deliberation	Parent–adolescent discussion; decision-making considerations
	Perceived Educational Value of the Game	Educational content	Appropriate content; relevance for parents; amount of information; quizzes
		Suggestions for content improvement	Navigation; more side-effect information; more disease-related information
	Enjoyment and Engagement in Gameplay	Enjoyment and fun	Fun; enjoyable; easy to play
		Favorite gameplay features	Blaster; enemies; monsters
	Avatar Realism and Representation	Avatar characteristics	Gender realism; desire for personality; avatars speaking
		Visual appearance of avatars	Color preferences; pixelation
	Visual Appeal and Design	Graphics and layout	Graphics; textures; color use
		Text presentation and pacing	Font size; readability; speed of text advancement
	Game Recommendation to Others	Recommendation behavior	Recommend to family; recommend to friends
**Healthcare Providers**			
	**Theme**	**Subtheme**	**Representative Codes**
	Patient Panel Characteristics	Demographics and social context	Diversity; language; immigrant families; underserved populations
		Clinical complexity	Chronic conditions; mental health concerns; neurodevelopmental conditions
	Parental Hesitancy and Vaccine Views	Parental concerns	Safety concerns; side effects; COVID-related skepticism
		Facilitators of acceptance	Trusting relationships; fewer injections
	Vaccine Misinformation	Common misconceptions	HPV vaccine only for girls; unnecessary without sexual activity; promotes sexual behavior
	Digital Tools and Workflow Integration	Game-based tools	Use during waiting time; multilingual needs
		Workflow considerations	Clinic flow; competing priorities; time constraints

## Data Availability

De-identified qualitative interview data are archived in OSF and available upon reasonable request. Public release will occur after completion of the full NIH-funded project and associated documentation.

## Clinicaltrial.gov Registration Number

NCT06162676.
